# Epidemiology of antiphospholipid syndrome: macro- and microvascular manifestations

**DOI:** 10.1093/rheumatology/kead571

**Published:** 2024-02-06

**Authors:** Pedro Gaspar, Savino Sciascia, Maria G Tektonidou

**Affiliations:** Internal Medicine Department, Hospital Santa Maria, Centro Hospitalar Universitário Lisboa Norte, Lisbon, Portugal; Instituto de Medicina Molecular João Lobo Antunes, Centro Académico de Medicina de Lisboa, Faculdade de Medicina, Universidade de Lisboa, Lisbon, Portugal; Department of Clinical and Biological Sciences, University Center of Excellence on Nephrologic, Rheumatologic and Rare Diseases (ERK-Net, ERN-ReConnect and RITA-ERN Member) with Nephrology and Dialysis Unit and Center of Immuno-Rheumatology and Rare Diseases (CMID), ASL Città Di Torino and University of Turin, Turin, Italy; First Department of Propaedeutic and Internal Medicine, Joint Academic Rheumatology Program, EULAR Centre of Excellence, National and Kapodistrian University of Athens, Medical School, Athens, Greece

**Keywords:** antiphospholipid syndrome, clinical manifestations, epidemiology, prevalence, incidence, macro- and microvascular events

## Abstract

Antiphospholipid syndrome (APS) is a systemic autoimmune disease characterized by thrombotic and non-thrombotic macro- and microvascular manifestations and pregnancy complications in the setting of persistent antiphospholipid antibodies (aPL), namely anticardiolipin antibodies, anti-β2 glycoprotein-I antibodies and lupus anticoagulant. Four decades after its first description, APS prevalence and incidence are still not completely understood due to the limited number of well-designed, population-based multi-ethnic studies. Furthermore, despite decades of efforts to standardise aPL immunoassays, considerable intraassay and interlaboratory variances in aPL measures still exist. Large multicentre APS cohorts have shown a 10-year survival of ∼91% and the presence of catastrophic APS occurs in about 1% of the entire population, associated with a 50% mortality rate. Clinically, any organ can be affected in the context of large, medium or small vessel (artery and/or vein) thrombosis. Macrovascular thrombosis is the hallmark of the disease and veins are more frequently affected than arteries. Deep vein thrombosis/pulmonary embolism thromboembolic disease is the most common APS manifestation, while stroke and transient ischaemic attack are the most frequent arterial thrombosis events. Myocardial infarction can also occur and contributes to increased mortality in APS. A minority of patients present with thrombosis affecting the intraabdominal organs, including the liver, spleen, small and large bowel, and the kidneys. Microvascular thrombosis, including APS nephropathy, chronic skin ulcers and livedoid vasculopathy represent a diagnostic challenge requiring histologic confirmation. In this narrative review we summarize the available evidence on APS epidemiology, focusing on the description of the prevalence of macro- and microvascular manifestations of the disease.

Rheumatology key messages

- In antiphospholipid syndrome (APS), thrombosis can affect any vessel (artery and/or vein) of any organ.
- Deep vein thrombosis/pulmonary embolism and stroke are the most common macrovascular APS manifestations.
- Most typical microvascular thrombosis includes livedoid vasculopathy, acute and chronic antiphospholipid antibody-nephropathy, and pulmonary haemorrhage.

## Introduction

Antiphospholipid syndrome (APS) is an acquired autoimmune thrombophilia characterized by pregnancy morbidity, venous and arterial thrombosis and/or microvascular manifestations in the presence of antiphospholipid antibodies (aPL) [1]. Although APS was recognized almost four decades ago, its prevalence and incidence, as well as the prevalence of aPL in different populations is still not fully defined due to the paucity of well-designed population-based multi-ethnic studies [2, 3]. Furthermore, despite decades of efforts for standardized aPL immunoassays, intraassay and interlaboratory variations in aPL measurements still exist, further challenging the available epidemiological data [4, 5].

In a population-based study of newly diagnosed APS patients between 2000 and 2015 in Olmsted County, Minnesota, the annual incidence of APS was 2.1 [95% confidence interval (CI): 1.4–2.8] per 100 000 population, and the estimated prevalence was 50 per 100 000 adults [6]. Conflicting reports exist regarding the prevalence of APS in males and females [6, 7], which can be attributed to different population characteristics among studies, e.g. primary *vs* secondary APS rates and racial/ethnic composition. In a recent review article including data from six population-based studies, the estimated incidence and prevalence for APS ranged from 1 to 2 and from 40 to 50 cases per 100 000 adults, respectively [2].

Regarding the prevalence of the clinical manifestations included in the APS classification criteria [1], data from the two largest multicentre registries so far, the EuroPhospholipid Project and the AntiPhospholipid Syndrome Alliance For Clinical Trials and International Networking (APS ACTION) registries, showed that venous thromboembolic disease, including deep venous thrombosis (DVT) and/or pulmonary embolism, and acute cerebrovascular events [stroke and transient ischaemic attacks (TIA)], are the most common thrombotic manifestations of APS [8, 9]. Additionally, several extra-criteria clinical features have been reported among APS patients with varying prevalence, including thrombocytopenia, APS nephropathy, valvular heart disease, skin ulcers and livedo reticularis [10]. Less than 1% of patients with APS develop catastrophic APS (CAPS), a life-threatening thromboembolic disease with a mortality rate of ∼50%, affecting three or more organ systems within 1 week [11, 12]. The survival probability at 10 years in the EuroPhospholipid registry including 1000 APS patients, the largest registry so far in APS, was 90.7% [13].

The laboratory criteria for APS include the presence of lupus anticoagulant (LAC) and medium-to-high titres of IgG and/or IgM anticardiolipin (aCL) and/or anti-ß2-glycoprotein I (aß2GPI) antibodies on two or more occasions at least 12 weeks apart [1]. Other aPL such as the IgA isotype of aCL and aß2GPI antibodies, IgG anti-domain 1 of aß2GPI antibodies, and IgG and/or IgM anti-phosphatidylserine/prothrombin antibodies have been described [14–17], not currently included in the Sydney or the new ACR/EULAR classification criteria for APS [1, 18]. The aPL subtype (LAC *vs* aCL *vs* aß2GPI antibodies), the presence of double or triple *vs* single positivity, the moderate-high *vs* low titre and the aPL persistence constitute the ‘aPL profile’. The high-risk aPL profile as defined by the EULAR recommendations for the management of APS in adults includes at least one of the following: (i) the presence of LAC (measured according to International Society for Thrombosis and Haemostasis guidelines) [5]; (ii) double (any combination of LAC, aCL or aß2GPI antibodies) or triple aPL positivity; and (iii) the presence of persistently high aPL titres [19].

aPL can be detected in 1–5% of healthy individuals and may also transiently occur during infections, mostly in low titres [20]. aPL have been observed to be more common in advanced age; however, no correlation was found between aPL positivity or their titres and development of APS manifestations in this group of healthy subjects [21]. A systematic literature review on the association between the presence of aPL and several vascular manifestations and/or pregnancy morbidity in the general population was published by the APS ACTION group [22]. APL positivity was found in 13%, 11%, 9.5% and 6% of patients with stroke, myocardial infarction, deep vein thrombosis and pregnancy morbidity, respectively [22]. However, a high heterogeneity in study design, aPL immunoassays and cut-off values for aPL positivity was observed among the included studies [22].

aPL are more frequently detected in subjects with other autoimmune disorders, mainly systemic lupus erythematosus (SLE). The prevalence of LAC, aCL and aß2GPI antibodies in SLE ranges from 15% to 34%, 12% to 44% and 10% to 19%, respectively [23]. Notably, ethnic and geographical variation in the prevalence of aPL has been described [20, 24], albeit evidence on this topic is scarce. Among others [25, 26], Molina and co-workers reported that 26% of Hispanics, 21% of Afro-Caribbeans, and 28% of African-American patients with SLE had at least one of the three aCL isotypes (IgG, IgM, IgA) [27]. A higher prevalence of IgG aCL was observed among Hispanic and African-Americans, whereas Afro-Caribbeans had more frequently an IgA aCL isotype [27]. Compared with Caucasian patients, the prevalence of LAC, aCL and aß2GPI antibodies was found to be relatively lower in Chinese patients with SLE (22.4%, 29% and 7.7%, respectively) [28]. However, the above studies were limited to patients with SLE [27, 28].

Here, we aimed at critically reviewing available evidence on the epidemiology of APS, with a specific focus on macro- and microvascular manifestations. We searched PubMed/Medline database using the following key terms: ‘antiphospholipid syndrome; epidemiology; prevalence; incidence; mortality; manifestations; symptoms; clinical characteristics’. We included only studies written in English and we focused our search on data obtained from cohorts with at least 100 thrombotic APS patients. Study and patient characteristics of the selected studies are summarised in Table 1. Table 2 provides data on the prevalence of macro- and microvascular manifestations reported in these studies.

**Table 1.** Study and patient characteristics in selected studies around the world^a^

Author, year	Cervera R, 2002 [8]	Sevim E, 2022 [9]	Qui Q, 2022 [36]	Shi H, 2017 [30]	Bertero MT, 2012 [31]	Ogata Y, 2021 [32]	Serrano R, 2020 [33]	Pengo V, 2009 [34]	Álvarez-López S, 2023 [37]	Mejía-Romero R, 2007 [35]
Type of study	Multicentre, prospective	Multicentre, prospective	Single centre, prospective	Single centre, retrospective	Multicentre, retrospective	Single centre, retrospective	Single centre, prospective	Multicentre, retrospective	Single centre, retrospective	Multicentre, prospective
Region	Europe (Euro-Phospholipid Project)	Europe, North America, Latin America, Asia (APS|ACTION registry)	China	China (APS-SH database)	Italy (Piedmont Cohort)	Japan	Spain (APS-CLINIC Registry)	Italy	Columbia	Colombia, Mexico, Ecuador
Total no. of patients	1000	642	383	252	217	168	160	160	103	100
Thrombotic patients, *n* (%)	879 (87.9)	568 (88.5)	>100^d^	190 (75.4)	171 (78.8)	>100^e^	117 (73.1)	160 (100.0)	103 (100.0)	100 (100.0)
Follow-up time	92 ± 75 (mth)	—	3 ± 2 (y)	—	—	10 (IQR 5–15) (y)	11 ± 6 (y)	—	—	—
Classification criteria	Wilson 1999	Miyakis 2006	Miyakis 2006	Miyakis 2006	Miyakis 2006	Miyakis 2006	Miyakis 2006	Miyakis 2006	Miyakis 2006	Miyakis 2006
**Demogaphics**										
Female sex, i (%)	820 (82.0)	393 (61.2)	269 (70.2)	216 (85.7)	162 (74.7)	144 (85.7)	126 (78.8)	113 (70.6)	86 (83.3)	92 (92.0)
F : M ratio	5:1	1.6:1		6:1	—	—	—	—	—	—
Age (disease onset)	34 ± 13 (y)	—	31 ± 12 (y)	—	44 ± 15 (y)	39 (range 30–55) (y)	39 ± 14 (y)	—	—	33 ± 11 (y)
Age (study entry)	42 ± 14 (y)	45 ± 13 (y)	38 ± 12 (y)	41 ± 12 (y)	43 ± 15 (y)	—	44 ± 14 (y)	41 ± 15 (y)	—	37 ± 12 (y)
Caucasian, *n* (%)	985 (98.5)	312 (66.2)	0	0	—	0	—	—	—	0
Latin American Mestizos, *n* (%)	0	75 (13.2)	0	0	—	0	—	—	—	100 (100.0)
Black, *n* (%)	5 (0.5)	17 (2.9)	0	0	—	0	—	—	—	0
Asian, *n* (%)	0 (0.0)	31 (5.5)	383 (100.0)	252 (100.0)	—	168 (100.0)	—	—	—	0
Other, *n* (%)	10 (1.0)	11 (1.9)	0	0	—	0	—	—	—	0
**Patients characteristics**										
Primary APS, *n* (%)	531 (53.1)	372 (65.5)	278 (72.6)	69 (27.4)	115 (53.0)	63 (37.5)	104 (65.0)	93 (58.1)	56 (54.3)	100 (100.0)
Secondary APS, *n* (%; %^b^)	469 (46.9)	196 (34.5)	105 (27.4)	183 (72.6)	102 (47.0)	105 (62.5)	56 (35.0)	67 (41.9)	47 (45.7)	0
SLE	363 (36.3; 77.4)	167 (29.4; 85.2)	93 (24.3; 88.6)	163 (64.7; 89.1)	60 (27.6; 58.8)	98 (58.3; 93.3)	36 (22.5; 58.9)	33 (49.3)	41 (39.8; 87.2)	—
Lupus-like syndrome	50 (5.0; 10.7)	—	—	16 (6.3; 9.8)	16 (7.3; 15.7)	—	14 (8.8; 25.0)	—	2 (1.9; 4.3)	—
Other	59 (5.9; 12.6)	—	12 (3.1; 11.4)	—	26 (12.0; 25.5)	8 (4.8; 7.6)	6 (3.8; 10.7)	34 (50.7)	4 (3.9; 8.5)	—
Catastrophic APS, *n* (%)	8 (0.8)	9 (1.6)	7 (1.8)	—	3 (1.4)	—	1 (0.6) | 4 (2.5)	4 (2.5)	4 (3.9)	0
Triple positivity, *n* (%)	—	278 (42.1)^c^	133 (34.7)	13 (5.2)	54 (24.9)	65 (38.7)	15 (29.4)	160 (100.0)	5 (4.9)	—

Data are presented as number (%) and mean ± standard deviation (SD) or range when appropriate.

a Only studies written in English were included with a sample size ≥100 adult patients with thrombotic APS.

b Proportion within the group.

c N = 660, total of aPL positive patients in the APS ACTION registry.

d Total number of thrombotic APS patients not specifically stated in the article (no. of patients: arterial, *n* = 127; venous, *n* = 164; obstetric, *n* = 142).

e Total number of thrombotic APS patients not specifically stated in the article (no. of patients: arterial, *n* = 108; venous, *n* = 53; obstetric, *n* = 50).

APS: antiphospholipid syndrome; F : M ratio: female to male ratio; IQR: interquartile range; mth: months; SLE: systemic lupus erythematosus; y: years.

**Table 2.** Prevalence of venous/arterial thrombotic events and microvascular manifestations in APS as reported in selected studies

Author, year	Cervera R, 2002 [8]	Sevim E, 2022 [9]	Qi Q, 2022 [36]	Shi H, 2017 [30]	Bertero MT, 2012 [31]	Ogata Y, 2021 [32]	Serrano R, 2020 [33]	Pengo V, 2009 [34]	Álvarez-López S, 2023 [37]	Mejía-Romero R, 2007 [35]
**Thrombotic manifestations, *n (%)*** ^a^	**Initial | Cumulative**	**Cumulative**	**Initial**	**Initial | Cumulative**	**Initial**	**Initial**	**Initial | Cumulative**	**Initial**	**Initial**	**Cumulative**
**Venous (any)** ^b^	**498 (49.8) | 731 (73.1)**	**327 (50.9)**	**237 (61.9)**	**106 (42.1) | 144 (57.1)**	**107 (49.3)**	**60 (35.7)**	**81 (50.6) | 92 (57.5)**	**76 (46.9)**	**90 (87.4)**	**38 (38.0)**
Thromboembolic disease	407 (40.7) | 564 (56.4)	300 (46.7)	192 (50.1)	102 (40.5) | 120 (47.6)	107 (49.3)	56 (33.3)	75 (46.9) | 80 (50.0)	70 (43.8)	80 (77.7)	26 (26.0)
Deep venous thrombosis	317 (31.7) | 423 (42.3)	224 (34.9)	110 (28.7)	90 (35.7) |103 (40.9)	81 (37.3)	39 (23.2)	59 (36.9) | 64 (40.0)	56 (35.0)	61 (59.2)	23 (23.0)
Lower limb	317 (31.7) | 389 (38.9)	217 (33.8)	110 (28.7)	90 (35.7) | 101 (40.1)	—	—	— | —	54 (33.8)	53 (51.5)	23 (23.0)
Upper limb	— | 34 (3.4)	7 (1.1)	—	— | 2 (0.8)	—	—	— | —	2 (1.3)	8 (7.8)	0 (0.0)
Pulmonary embolism	90 (9.0) | 141 (14.1)	76 (11.8)	82 (21.4)	12 (4.8) | 17 (6.7)	26 (12.0)	17 (10.1)	16 (10.0) |16 (10.0)	14 (8.8)	19 (18.4)	3 (3.0)
Superficial thrombophlebitis	91 (9.1) | 117 (11.7)	—	—	4 (1.6) | 12 (4.8)	—	2 (1.2)	— | —	—	—	7 (7.0)
Visceral venous thrombosis	— | 7 (0.7)	8 (1.2)	20 (5.2)	— | 1 (0.4)	—	—	6 (3.8) |6 (3.8)	1 (0.6)	—	0 (0.0)
Mesenteric	— | —	—	—	— | —	—	—	— | —^f^	1 (0.6)	—	0
Hepatic vein	— | 7 (0.7)	—	—	— | 1 (0.4)	—	—	6 (3.8) | 6 (3.8)	—	—	0
Splenic vein	— | —	—	—	— | —	—	—	— | —^f^	—	—	0
Renal vein	— | —^c^	—	—	— | —^c^	—	—	— | —^c^	—	—	0
Central sinus venous thrombosis	— | 7 (0.7)	13 (2.0)	19 (5.0)	— | 2 (0.8)	—	—	— | —	—	5 (4.9)	3 (3.0)
Retinal vein thrombosis	— | 9 (0.9)	6 (0.9)	6 (1.6)	— | 5 (2.0)	—	2 (1.2)	— | —	2 (1.3)	—	—
Other veins	— | 27 (2.7)	—	—	— | 4 (1.6)	—	—	— | —	2 (1.3)	5 (4.9)	2 (2.0)
Subclavian	— | 18 (1.8)	—	—	— | 1 (0.4)	—	—	— | —	—	—	2 (2.0)
Jugular	— | 9 (0.9)	—	—	— | 3 (1.2)	—	—	— | —	1 (0.6)	—	—
Vena cava	— | —	—	—	— | —	—	—	— | —	1 (0.6)	—	—
**Arterial (any)** ^b^	**279 (27.9) | 551 (55.1)**	**311 (48.4)**	**140 (36.6)**	**67 (26.6) | 108 (42.9)**	**63 (29.0)**	**109 (64.9)**	**100 (62.3) | 121 (75.6)**	**69 (43.1)**	**34 (33.0)**	**26 (26.0)**
Ischaemic stroke	131 (13.1) | 198 (19.8)	165 (25.7)	70 (18.3)	42 (16.7) | 60 (23.8)	53 (24.4)	92 (54.8)	36 (22.5) | 47 (29.4)	27 (16.9)	25 (24.3)	18 (18.0)
Transient ischaemic attack	70 (7.0) | 111 (11.1)	69 (10.7)	—	11 (4.4) | 11 (4.4)	—	—	8 (5.0) | 10 (6.3)	15 (9.4)	—	—
Myocardial infarction	28 (2.8) | 55 (5.5)	31 (4.8)	18 (4.7)^d^	3 (1.2) | 5 (2.0)	10 (4.6)	6 (3.6)^d^	9 (5.6) | 10 (6.3)^d^	8 (5.0)	2 (1.9)	3 (3.0)
Coronary bypass thrombosis	— | 11 (1.1)	—	—	— | 1 (0.4)	—	—	— | —	—	—	—
Peripheral artery thrombosis	19 (1.9) | 103 (10.3)	30 (4.7)		11 (4.4) | 22 (8.7)	—	5 (3.0)	10 (6.3) | 12 (7.5)	15 (9.4)	7 (6.8)	4 (4.0)
Lower limb	— | 43 (4.3)	—	23 (6.0)	— | 6 (2.4)	—	5 (3.0)	— | —	15 (9.4)	7 (6.8)	1 (1.0)
Upper limb	— | 27 (2.7)	—	—	— | 2 (0.8)	—	—	— | —	—	—	2 (2.0)
Digital gangene	19 (1.9) | 33 (3.3)	—	—	11 (4.4) | 14 (5.6)	—	—	— | —	—	—	1 (1.0)
Visceral arterial thrombosis	— | 58 (5.8)	11 (1.7)	20 (5.2)	— | 15 (6.0)	—	4 (2.4)	17 (10.6) | 19 (11.9)	3 (1.9)	—	1 (1.0)
Mesenteric artery	— | 15 (1.5)	—	—	— | 2 (0.8)	—	3 (1.8)	3 (1.9) | 3 (1.9)^g^	1 (0.6)	—	1 (1.0)
Splenic artery	— | 11 (1.1)	—	—	— | 3 (1.2)	—	—	— | —	—	—	0
Pancreatic artery	— | 5 (0.5)	—	—	— | 0 (0.0)	—	—	— | —	—	—	0
Hepatic artery	— | —	—	—	— | —	—	—	— | —	—	—	—
Renal artery	— | 27 (2.7)^d^	—	—	— | 10 (4.0)^e^	—	1 (0.6)	14 (8.8) | 16 (10.0)^e^	2 (1.3)	—	—
Retinal artery thrombosis	— | 15 (1.5)	5 (0.8)	9 (2.3)	— | 4 (1.6)	—	2 (1.2)	20 (12.5) | 23 (14.4)^h^	1 (0.6)	—	—
**Other**										
Amaurosis fugax	28 (2.8) | 54 (5.4)	—	—	2 (0.8) | 3 (1.2)	—	—	— | —	—	—	—
Adrenal insufficiency	— | 4 (0.4)	—	—	— | 0 (0.0)	—	—	— | —	—	—	1 (1.0)
**Microvascular (biopsy proven)** ^b^	**39 (3.9) | 115 (11.5)**	**74 (11.5)**	**19 (4.9)**	**12 (4.8) | 18 (7.1)**	**Not stated**	**Not stated**	**Not stated**	**3 (1.9)**	**4 (3.9)**	**9 (9.0)**
Skin ulcers	39 (3.9) | 55 (5.5)	47 (7.3)	2 (0.5)	12 (4.8) | 11 (4.4)	—	—	— | —	—	4 (3.9)	3 (3.0)
Cutaneous necrosis	— | 21 (2.1)	9 (1.4)	—	— | 3 (1.2)	—	—	— | —	3 (1.9)	—	1 (1.0)
Avascular necrosis	— | 24 (2.4)	—	—	— | 4 (1.6)	—	—	— | —	—	—	0
Kidney	—^c^	15 (2.3)	17 (4.4)	—^c^	—	—	—^c^	—	—	—
Pulmonary	— | 15 (1.5)	3 (0.5)	—	— | 0 (0.0)	—	—	— | —	—	—	5 (5.0)

Data are presented as number (%) and mean ± standard deviation (SD) or range when appropriate.

a Number (%) of patients.

b The number of affected patients is calculated from the sum of the number of clinical manifestations. The percentage is calculated for the entire population. Data might differ from original works.

c Included in renal artery thrombosis.

d It is not stated whether this includes acute events and/or chronic coronary artery disease.

e Includes arterial (renal infarction, renal artery thrombosis), venous (renal vein thrombosis) and microvascular events (glomerular thrombosis).

f Included in visceral artery thrombosis.

g Includes mesenteric and splenic arterial thrombosis, venous thrombosis, thrombotic microangiopathy and angioplasty thrombosis.

h Includes central retinal artery and vein thrombosis, and ischemic optic neuritis.

APS: antiphospholipid syndrome; F : M ratio: female to male ratio; mth: months; SLE: systemic lupus erythematosus; y: years.

## Macrovascular manifestations in APS

Venous and/or arterial thrombosis is the hallmark of the presentation of APS. Any vessel and/or organ system can be affected either as single or multiple events, occurring simultaneously or sequentially [29]. The wide spectrum of APS thrombotic manifestations in APS echoes the great variability in the type of thrombosis (arterial/venous), location and vessel size (macro- and microvascular).

### Venous thrombosis

Venous thrombosis, either as initial or recurrent events, is more prevalent than arterial thrombosis, with estimates ranging from ∼40% to 50% [9, 30–35] to >60% of patients [8, 36, 37]. The demographic characteristics (age, sex and race/ethnicity), disease duration, co-existence of risk factors for venous thrombosis (trauma, surgery, immobilization, oestrogen use or obesity) and the access to a health care system, modulate how APS manifests worldwide.

Deep venous thrombosis (DVT) is the most frequent venous thrombosis manifestation in APS, affecting 30–40% of patients [8, 9, 30, 31, 33, 34, 36] (Table 2). The deep veins of the lower limbs are the most common sites of thrombosis, affecting more than one-third of patients in most regional and international cohorts [8, 9, 30, 34, 37]. The upper limbs can also be affected, although with a much lower prevalence (∼1% to 3%). Less commonly (<1%), other sites of venous thrombosis include subclavian, jugular and superior and/or inferior vena cava [8, 30, 34]. DVT is arguably very unlike to be life-threatening, nevertheless, post-thrombotic syndrome, encompassing venous vascular insufficiency to chronic ulcers and cutaneous trophic lesions, can develop in >40% of thrombotic APS patients [38] and has a significant impact on health-related quality of life [39]. Superficial thrombophlebitis, although not included in the APS classification criteria, may occur in up to 10% of patients [8, 30, 35].

Pulmonary embolism is the most frequent pulmonary manifestation in APS, affecting 11% to 20% of individuals [8, 9, 31, 36, 37] and can be the presenting manifestation in about 9% to 12% of patients [8, 9, 31–34]. It may occur as an isolated entity, but more often follows a lower limb DVT [40]. The prevalence of DVT/pulmonary embolism thromboembolic disease ranges from 40% to 50% in large registries [8, 9, 30, 31, 34, 36]. Despite adequate treatment, APS patients experience recurrent thrombotic events [34, 41] and the pattern of initial clinical manifestations seems to be preserved with regard to the second event [42–44]. This may explain the increase in the prevalence of pulmonary embolism from 5.2% to almost 12% during a 10-year follow-up in the Euro-Phospholipid cohort [41]. Chronic thromboembolic pulmonary hypertension (CTEPH) results from the incomplete resolution of pulmonary embolism and the development of chronic obstructions within the pulmonary artery beds [45]. It is a rare complication of symptomatic pulmonary embolism with a cumulative incidence of 0.79% at two years follow-up in the general population [46], but a higher risk is observed in those with unprovoked and recurrent pulmonary embolism (multivariate odds ratio: 5.70 and 19.0, respectively) [47], as is the case of most APS patients. Accordingly, in a retrospective study including 297 consecutive patients with CTEPH, previous history of pulmonary embolism was more frequent among APS (96%) compared with non-APS patients (66%) [48]. Although scarcely studied, CTEPH seems to affect ∼4% to 5% of thrombotic APS patients [30, 36, 49–51].

Cerebral vein thrombosis (CVT), including the thrombotic occlusion of the dural venous sinus and/or cerebral veins, represents only 0.5–0.7% of the cerebral vascular complications in the general population [52]. Being such a rare manifestation, its exact prevalence in thrombotic APS is difficult to be determined. In the Euro-Phospholipid Project including 1000 APS subjects [8], only seven (0.7%) patients presented with a CVT. Further studies, including the APS ACTION registry, report a prevalence of around 2% [9] to 5% [36, 37] in APS. On the other hand, APS contributes to a significant proportion (∼6% to 17%) of CVT cases in large studies in the general population [53, 54]. Recently, Jerez-Lienas *et al.* [55] analysed the data from a series of 27 APS patients with CVT from three university hospitals, and a systematic literature review of CVT cases in APS. The lateral venous sinuses, including transverse and sigmoid sinuses, were the most frequently involved, followed by the superior sagittal sinus, while cerebral cortical veins thrombosis was less frequent (14% of cases) [55]. When present, CVT can be the first APS manifestation in almost 75% of patients [55], while headache is the most common symptom at presentation [52, 55, 56]. Interestingly, it can be the only symptom in >40% of APS-related cases [55]. Any change from the usual headache pattern and especially the co-existence with new neurologic symptoms, should prompt a careful imaging evaluation with brain computed tomography (CT) and/or magnetic resonance imaging (MRI) [57].

Ocular manifestations in APS may involve the anterior and posterior eye segments, visual pathways, or the central nervous system [58, 59]. Asymptomatic abnormalities can be present in >80% of patients [60, 61], but overt thromboses are rarer. Clinical manifestations include monocular or binocular blurring of vision, amaurosis fugax, transient diplopia and transient visual field losses [60, 62]. The posterior segment of the eye is the most frequently affected [59, 60]. This includes central or branched retinal vein occlusions [62] that can affect ∼1% to 2% of patients with APS [8, 9, 30, 32, 34, 36]. In APS, venous occlusions display a distinct pattern of retinal affectation compared with those occurring in the context of arteriosclerosis-related vasculopathy [63].

Splanchnic venous thrombosis occurs in about 1% of patients with APS [8, 9, 34]. A wide spectrum of hepatic complications has been associated with the presence of aPL, ranging from non-thrombotic manifestations to overt macro- and/or microthrombosis [64–67]. Hepatic vein thrombosis was described in 0.7% of APS patients in the Euro-Phospholipid Registry [8] and may result in Budd–Chiari syndrome [64–68], ultimately leading to portal hypertension and cirrhosis. Usually manifested with abdominal pain, hepatomegaly and ascites, its clinical presentation may range from almost asymptomatic to fulminant liver failure [68]. Notably, it can be the first manifestation of APS, particularly in the setting of primary APS [68]. Splenic and mesenteric vein thrombosis are rare events (<1%) and can occur independently [30, 34] or in the context of portal vein thrombosis [69, 70] and/or of concomitant occlusion of several intra-abdominal vessels, including renal thrombosis [71, 72]. Portal vein thrombosis can also be the first thrombotic manifestation of APS [73].

Renal vein thrombosis (RVT) is a rare complication of APS occurring in about 1% of patients and usually unilaterally [74–76], although more rarely, bilateral RVT has been also reported [66, 75]. The precise prevalence of RVT in the context of APS remains uncertain (Table 2). A significant association with the presence of aPL has been demonstrated in patients with SLE [66, 77], even though RVT might be also a complication of nephrotic syndrome in SLE [78, 79]. Lumbar pain, haematuria and new-onset or worsening of proteinuria should raise a suspicion for this rare manifestation [79].

Globally, the prevalence of intra-abdominal organ thrombosis, either venous, arterial and/or microvascular thrombosis, is difficult to be determined due to their rarity but also due to a high heterogeneity in reported data from different APS cohorts (Table 2). Mixed data from both arterial and venous thrombosis are often presented by cohort studies; for example, renal manifestations may include composite data from renal vein or artery thrombosis, renal infarction or APS nephropathy [8, 30, 33]. In some studies, abdominal organ manifestations (such as splanchnic organ arterial or venous thrombosis) and renal involvement may be pooled together [33], or they are stated without specifying the involved organ [36]. A standardized protocol of how we collect and classify data is needed for a better estimation of the epidemiology of abdominal manifestations in APS.

### Arterial thrombosis

Even though arterial manifestations are less common than venous thrombosis events in APS, they are usually more severe and potentially life-threatening. In the Euro-Phospholipid Project registry, stroke and myocardial infarction were the most common causes of death (22.5% of cases) [13]. Despite the established association between ischaemic arterial events and aPL [80, 81], the role of traditional cardiovascular disease risk factors such as age, sex, smoking, diabetes and high blood pressure is increasingly recognized in APS [82]. Regarding the impact of race/ethnicity, arterial thrombosis was found to be more common than venous thrombosis in some APS registries from Asia [32, 83] (Table 2).

Acute cerebrovascular disease, including ischaemic stroke and TIA, is the most common arterial thrombotic manifestation in APS [29]. The prevalence of stroke in large cohorts ranges between 20 and 30% [8, 9, 30, 31, 33, 36], and the reported prevalence of TIA is ∼10% [8, 9, 33, 34]. On the other hand, in a systematic literature review of studies in the general population, APS was the cause of acute ischaemic stroke in 20% of patients under 45 years of age [84]. Additionally, the estimated prevalence of aPL was 17.2% and 11.7% among patients with stroke or TIA, respectively, and has been associated with a five-fold higher risk for stroke or TIA compared with aPL-negative individuals in the general population [85]. *In situ* thrombosis and cardioembolic disease, either due to left-sided cardiac valve abnormalities (e.g. Libman–Sacks endocarditis) or, rarely, intracardiac thrombi, have been implicated in the pathogenesis of the above cerebrovascular ischaemic events [84, 86]. Infarcts of various sizes, including large infarcts mainly affecting the middle cerebral artery and small cortical infarcts, or lacunar infarcts, as well as hyperintense white matter foci, are the most common finding in brain imaging studies in APS [87, 88]. The clinical presentation of APS-related cerebrovascular disease depends on the location and size of arterial thrombosis. Stroke has been documented as one of the main causes of mortality in APS (up to 18% of deaths) [13, 89, 90], and the most common cause of permanent disability (e.g. hemiplegia, hemiparesis) reported in up to 20% of cases [50].

Small vessel cerebrovascular disease can also occur in ∼10% of APS patients, leading to lacunar infarctions defined as small subcortical lesions (<15 mm in diameter) on brain imaging [87]. Multifocal white matter lesions are a common finding on brain MRI in APS patients and have been significantly associated with the presence of cognitive deficits [91, 92]. Sneddon’s syndrome, a slowly progressive noninflammatory thrombotic vasculopathy characterized by livedo racemosa (a violaceous symmetric net-like widespread pattern with characteristic irregular ‘broken’ appearance [93]) and recurrent cerebrovascular events [94] has been also associated with the presence of aPL [94], although its exact prevalence in APS is not known due to the scarcity of reports.

Even though cardiac valve abnormalities can be detected by cardiac transthoracic or transoesophageal ultrasound in more than one-third of APS patients [33, 95], overt thrombotic cardiac manifestations are relatively rare [51]. Myocardial infarction in APS is observed in ∼2–5.5% of patients [8, 9, 30, 31, 34–37], and often presents as the first manifestation of the disease (73%) [96]. Myocardial ischaemic events can result from coronary artery thrombosis with or without underlying atherosclerosis, or a microvascular injury detected by cardiac MRI [97, 98] which is also often associated with myocardial dysfunction in APS [99]. Of note, myocardial infarction with nonobstructive coronary arteries was the main finding in many cases [96, 100, 101], particularly among younger individuals [100, 102]. Coronary bypass re-thrombosis has also been rarely reported (<1%) [8, 30] (Table 2). Moreover, APS is characterized by accelerated rates of subclinical atherosclerosis and arterial stiffness to a degree comparable to diabetes mellitus [103, 104]. Evidence from experimental studies has shown an interplay among aPL-mediated thrombotic, inflammatory and atherogenesis mechanisms in the pathogenesis of cardiovascular events in APS [82]. Clinical studies have also shown a higher prevalence of the traditional cardiovascular risk factors in patients with APS *vs* healthy individuals and other chronic high cardiovascular risk disorders, such as rheumatoid arthritis and diabetes mellitus [105, 106]. These data support the need for aggressive management of cardiovascular risk factors in this population [107] as it was also underlined by the recent EULAR recommendations for the management of cardiovascular risk in rheumatic and musculoskeletal diseases, including SLE and APS [108]. Intracardiac thrombus is a rare (∼0.5% to 2%) but potentially life-threatening manifestation of APS [8, 9, 30] and may occur at any cardiac chamber in isolation or simultaneously [109].

Peripheral artery disease is far less common than peripheral venous thrombosis in APS, affecting 5% to 12% of patients (Table 2). Most studies describe peripheral artery disease of mainly lower limbs [32, 34–37]. In studies also reporting upper limb ischaemia cases, lower limbs involvement is more prevalent (∼27% to 42% of cases) [8, 30]. In a recent systematic review and meta-analysis aiming to evaluate the clinical relevance of aPL in patients with lower extremity artery disease, the pooled prevalence of IgG aCL and LAC was 12% and 13.3%, respectively [110], and LAC was more frequent in patients with failed *vs* those with successful revascularisation (35.8% *vs* 15.8%) [110]. Digital gangrene, often bilateral, can affect the upper and lower extremities [111, 112]. In most registries, it occurs in ∼3% to 6% of APS patients [8, 30] and often in the context of catastrophic APS [12, 66, 113–115].

Central and branched retinal artery occlusion has been described in APS [59, 60, 62] in 1% to 2% of patients [8, 9, 30, 32, 36]. In rarer cases, occlusion of the cilioretinal artery can lead to diffuse or localised choroidal infarction [116, 117]. Diffuse peripheral ischaemic retinopathy with neovascularisation can also develop [62, 118]. Non-arteritic ischaemic optic neuropathy occurring due to decreased blood supply to the optic nerve head is another rare APS ocular manifestation [119]. Focal CNS ischaemia along the visual pathway or cerebral cortex can lead to visual symptoms with unremarkable fundoscopic findings [62].

Arterial thrombosis of intra-abdominal organs occurs more frequently in APS (up to 6%) compared with intra-abdominal venous thrombosis [8, 30, 36]. Mesenteric artery thrombosis is present in ∼0.6% to 2% of APS patients [8, 30, 32, 34], either in the context of catastrophic APS [12, 66] or as isolated cases [120, 121], and even as the first APS presentation [121]. It can lead to small and/or large-bowel perforation that warrants emergency management [122]. Chronic abdominal angina has also been described in APS [123]. Splenic infarcts may occur in ∼1% of APS patients [8, 30, 83] and often co-exists with other abdominal organs ischaemia [121, 123]. Interestingly, APS has been recognized as one of the major causes of splenic infarction in the general population [124]. Auto splenectomy or functional asplenia is a rare complication [125, 126]. Hepatic and pancreatic infarctions occur very rarely (<1%) [8]. Anecdotal cases of acute oesophageal necrosis [127] and gangrene of the stomach [128] associated with APS have also been reported.

Renal artery thrombosis has been reported in about 1% of APS patients [32, 34, 83] (Table 2). It can be unilateral [129, 130] or bilateral [131], isolated [129, 130], or in co-existence with other intra-abdominal organ ischaemia manifestations [71], including aortic thrombosis [132]. Both distal and proximal renal artery stenosis with *in situ* thrombosis, and embolic occlusion have been described [75, 133]. Renal artery thrombosis may present as renal infarction, acute renal failure or slowly progressive chronic renal failure, but most commonly as uncontrolled or new onset severe hypertension [74, 129, 130, 133].

## Microvascular manifestations in APS

Thrombosis of the microvasculature may also occur as part of the APS clinical manifestations, either before or after the development of macrovascular manifestations. The diagnosis of microvascular manifestations may be challenging because, in addition to their rarity, current classification criteria require, along with appropriate imaging, a confirmation with histopathology studies [1]. A high heterogeneity in the methodology of clinical studies reporting microthrombotic events still exists, further challenging the accuracy of available epidemiological data; while some studies follow the APS classification criteria for microvascular events [8, 9, 30, 35], this does not hold true for others [36, 37, 134].

Microvascular thrombosis may occur at any organ system in APS. This is often part of CAPS presentation [12, 135], although acute isolated [136] and chronic manifestations may also occur [137]. Globally, microthrombosis manifestations can be found in up to 12% of APS patients [8, 9, 30] (Table 2). The kidneys and skin seem to be the most frequently affected, but are also the more readily accessible for biopsy confirmation.

The Renal Pathology Subcommittee for the 2023 ACR/EULAR classification criteria for APS concluded in the most accepted terminology for both acute and chronic renal histologic lesions of aPL-associated nephropathy, the so-called APS nephropathy [138], including the presence of acute lesions of thrombotic microangiopathy (fibrin thrombi in glomeruli and/or arterioles) and/or various chronic renal small-vessel lesions such as fibrous intimal hyperplasia, organized glomerular or arterial/arteriolar microthrombi, fibrous arterial/arteriolar occlusion, and focal cortical atrophy [138]. Common clinical and laboratory manifestations include new onset or worsening of previously diagnosed hypertension, microscopic haematuria, mild to nephrotic level proteinuria and variable degrees of renal insufficiency [139–141]. Acute APS-related thrombotic microangiopathy, probably the most severe and challenging form of APS nephropathy, may be limited to the kidneys, or be accompanied by clinical features of microangiopathic haemolytic anaemia, thrombocytopenia, and ischemic extra-renal end-organ injury, including the central nervous system, cardiovascular and respiratory systems, and the gastrointestinal tract [141, 142]. In these cases, other causes of thrombotic microangiopathy should be excluded such as complement-mediated [atypical haemolytic uremic syndrome (aHUS)], ADAMTS13-mediated [thrombotic thrombocytemic purpura (TTP)], pregnancy-associated [haemolysis, elevated liver enzymes, and low platelet count (HELLP) syndrome], other autoimmune disorders-associated (systemic sclerosis), and infection- or drug-induced (immunosuppressives, chemotherapy, antibiotics) thrombotic microangiopathy [12, 142].

APS nephropathy histologic lesions have been observed among patients with primary APS, CAPS, but also aPL-positive SLE patients with or without overt thrombotic APS [139, 141, 143–145]. Importantly, the absence of immune complexes is essential for the identification of APS nephropathy, particularly in the setting of APS related to other connective tissue disease, mainly SLE [10, 139]. The prevalence of APS nephropathy is difficult to determine and is probably underestimated in primary APS where kidney biopsies are not systematically performed. Estimated prevalence of APS nephropathy ranges from ∼9% to 30% in primary APS [141, 146], to >30% in aPL-positive SLE patients [139, 145]. In the task force report on non-criteria manifestations, the overall quality of evidence for APS nephropathy was graded as moderate [147], hence it was included in the new classification criteria for definite APS [18].

In the skin, microvascular thrombosis may manifest as livedoid vasculopathy and skin ulcerations [148]. Livedoid vasculopathy is a rare, chronic and recurrent occlusive disorder in the microcirculation of dermal vessels [149]. Clinically, it is characterized by painful ulceration usually located in the distal parts of the lower extremities, followed by healing as porcelain-white, atrophic scars. Histological characteristics include segmental hyalinization and intravascular fibrin deposition with or without co-existent thrombi [149]. It is highly associated with hypercoagulable states and has been described in association with aPL [150–153]. However, its prevalence in APS is not well established. Skin ulcerations are present in 4% to 7% of APS patients [8, 9, 30, 37, 154]. Skin ulcerations represent a diagnostic challenge because they can be the manifestation of several APS-related macro- and/or micro- pathologic conditions (e.g. post-DVT venous insufficiency; chronic arterial peripheral disease; APS microvasculopathy) or might also be associated with concomitant comorbid conditions (e.g. diabetes mellitus neuropathy; vasculitic lesions in patients with co-existent systemic) [66, 154]. Rarely, extensive cutaneous necrosis can be the sole manifestation of APS [154, 155].

Livedo reticularis is the most common dermatological manifestation in APS present in >20% of patients [8, 9, 154]. Although its pathogenesis does not necessarily involve thrombosis of the microvasculature [93], livedo reticularis may be a prognostic marker of a more severe APS phenotype characterized by arterial and/or microvascular manifestations [91, 154, 156, 157]. The quality of evidence supporting its inclusion in the APS classification criteria was graded as moderate in a systematic literature review by Abreu *et al.* [147]. A recent meta-analysis including 4810 SLE patients showed that the overall odds ratio for livedo reticularis in aPL-positive patients compared with aPL-negative patients was 2.91 (95% CI 2.17–3.90) [158]. Due to its limited specificity, livedo reticularis was not included in the new ACR/EULAR classification criteria for APS [18].

Biopsy is difficult to be performed in some small-vessel manifestations in APS and the diagnosis often relies on other diagnostic procedures, including imaging studies. Avascular necrosis (AVN) of the bone is a skeletal disease characterized by the death of bone cellular components as a result of interruption of the blood supply [159]. AVN affects ∼2% of APS patients [8, 30, 160]. The most common site of AVN is the femoral head and pain is almost always the presenting symptom [159], although asymptomatic AVN was also detected by bone MRI in a prospective study of patients with primary APS [161].

Pulmonary microvascular manifestations in APS include widespread thrombotic occlusions affecting the small pulmonary arteries or alveolar capillary lumens, with or without evidence of pulmonary capillaritis [66, 162–165]. In two large multicentre registries, pulmonary microthrombosis was reported in 0.5–1.5% of patients [8, 9]. Clinical presentation ranges from dyspnoea, cough and haemoptysis to variable degrees of respiratory failure. It can also manifest as diffuse alveolar haemorrhage [162] and/or acute respiratory distress syndrome [163, 164], both rare but devastating manifestations of APS (0.7%) [8].

## The adding value of the new ACR/EULAR classification criteria

The 2023 ACR/EULAR classification criteria for APS were published in October 2023 [18]. They updated the 2006 Sydney criteria by redefining some of the previously included clinical manifestations and incorporating some other aPL-related manifestations that were not previously included [18]. The new ACR/EULAR criteria, following a rigorous data-driven and expert-based methodology, have higher specificity (99% *vs* 86%) compared with the 2006 revised Sapporo classification criteria and require the presence of at least three points from the clinical and three points from laboratory domains for the APS classification. In a hierarchically clustered, weighted and risk-stratified manner, the ACR/EULAR classification criteria for APS now weight venous and arterial thrombosis according to the presence of a specified high-risk profile for venous thromboembolism or cardiovascular disease, respectively. Another improvement was the addition of well-defined microvascular domain items including *livedo racemosa*, livedoid vasculopathy, acute/chronic aPL-nephropathy, pulmonary haemorrhage, myocardial infarction with nonobstructive coronary arteries and adrenal haemorrhage. Additionally, cardiac valve disease (thickening and vegetation) and thrombocytopenia are also now considered. The incorporation of several of the previously classified as ‘non-criteria’ manifestations aimed to capture and quantify the magnitude and heterogeneity of APS manifestations.

## Conclusion

Despite our progress in APS understanding over the past decades, we still have a lot to learn about the epidemiology of this condition, and there is still a considerable knowledge gap mainly due to methodological aspects. Firstly, the attribution of some clinical events to the presence of aPL exclusively can be challenging because the pathogenesis of thrombosis (both in macro and micro vasculature) can be related to different than, or additional to, aPL factors. How to account for multiple factorial triggered events when evaluating the prevalence and incidence of APS remains an open question. Secondly, the heterogeneity in terms of clinical and aPL profiles among studies and among different ethnic groups further challenges their estimation. Finally, the classification criteria applied so far might not have adequately accounted for the entire clinical spectrum of APS, leading to underestimation of some of the clinical features of the syndrome. While not meant for diagnostic purposes, the new ACR/EULAR classification criteria have the potential to improve our ability in identifying cases suspected of APS, especially when clinical presentation goes beyond macrothrombosis. Advances in APS research will allow a better estimation of the geo-epidemiology of this complex disorder.

## Data Availability

All data used to support this article is provided in the Tables.
